# Resuscitation Leadership Education: A Needs Assessment of Emergency Medicine Residencies

**DOI:** 10.5811/westjem.47285

**Published:** 2025-12-26

**Authors:** Michael Sobin, Brett Todd, Nai-Wei Chen, Danielle Turner-Lawrence

**Affiliations:** * Corewell Health William Beaumont University Hospital, Department of Emergency Medicine, Royal Oak, Michigan; † University of Missouri School of Medicine, Department of Biomedical Informatics, Biostatistics and Medical Epidemiology, Columbia, Missouri

## Abstract

**Introduction:**

Effective resuscitation leadership is a critical competency for emergency physicians, with evidence correlating strong leadership with improved team performance and patient outcomes during resuscitations. Despite its importance, the extent and nature of structured resuscitation leadership education in emergency medicine (EM) residency training remains unclear.

**Methods:**

We conducted a voluntary, anonymous, needs assessment survey of United States (US) EM residency programs between August–October 2021. The survey assessed for the presence, content, and methods of formal resuscitation leadership curricula within these programs. We used descriptive statistics to analyze responses.

**Results:**

Of the 261 US EM residency programs invited to participate, 80 responded (30.7%). Nineteen programs (23.8%) reported offering resuscitation leadership training through formal curricula, with considerable variation in both educational methods and content. Additionally, 68.4% of responding programs offered external generalized leadership development opportunities through partnerships with hospitals, universities, community organizations, and research entities.

**Conclusion:**

A minority of surveyed US EM residency programs incorporate formal resuscitation leadership training into their curricula with significant variance in curricular content and educational methods. Given the critical role of resuscitation leadership in EM, our findings highlight the need for further research to evaluate the effectiveness of existing curricula and educational approaches.

## INTRODUCTION

Resuscitation leadership is a necessary skill for emergency physicians, with multiple studies demonstrating its significant impact on patient outcomes in high-pressure resuscitation scenarios.^1^–^3^ Strong leadership has been shown to enhance team performance during cardiopulmonary resuscitation.^4^ Similarly, key leadership competencies such as role delegation, task management, and effective communication have been found to influence both team performance and patient survival during resuscitation efforts.^2^ Cooper and Wakelam highlighted a positive correlation between physician leadership and improved in-hospital resuscitation outcomes.^5^ Notably, Chan et al demonstrated that the presence of actively engaged physician leaders during in-hospital resuscitations was significantly associated with improved patient survival.^3^ Additionally, a systematic review by Restivo et al demonstrated a positive link between leadership interventions and healthcare outcomes.^6^

Given the crucial role of resuscitation leadership in optimizing patient outcomes, it is essential that emergency medicine (EM) residents receive adequate training to develop into competent resuscitation leaders. The Accreditation Council for Graduate Medical Education (ACGME), in partnership with the American Board of Emergency Medicine, Association of American Medical Colleges, Council of Residency Directors in Emergency Medicine, Emergency Medicine Residents’ Association (EMRA), and the Review Committee for Emergency Medicine recognized the importance of leadership development when designing the EM Milestones. Consequently, leadership skills were embedded as essential competencies required for the successful completion of EM residency.^7^ These milestones encompass a wide range of skills, including emergency stabilization, multitasking, system navigation, professional behavior, and effective communication. However, it is unclear whether the implementation of these milestones has translated into effective leadership instruction for EM residents.^8^ Notably, in early 2025 the ACGME proposed additional requirements specifically mandating that EM residents demonstrate competence in leading a variety of resuscitations. This development reflects increasing national attention to resuscitation leadership as a core skill and underscores the need to better understand how current EM residency programs are training residents to meet this expectation.

Despite the clear need for leadership training, gaps in leadership development across GME persist. Over the past several decades, scholarly work has underscored the need for well-designed, competency-based leadership training for medical trainees.^9^ However, despite calls for action and the recognized impact of resuscitation leadership on patient outcomes, there remains a scarcity of well-researched and widely implemented leadership curricula.^10^–^13^ Additionally, EM residents have reported limited structured opportunities for leadership development during their training, with observation and non-specific feedback from faculty and peers serving as the most frequently cited leadership-learning strategies.^14^ Although EM residents participate in a high volume of clinical resuscitations, there is no current evidence evaluating whether EM graduates feel adequately prepared to lead resuscitations based solely on this experiential learning. No studies have assessed whether informal, experience-based instruction sufficiently develops the complex leadership competencies required in high-stakes resuscitative care.

Few EM resuscitation leadership resident educational approaches have been published.^15^,^16^ Additionally, the delivery of formal curricula focused on resuscitation leadership within EM residency programs remains unclear, as do the methods and content of existing programs.^1^,^8^,^11^,^12^ Limited research in medical education suggests that effective training may include explicit instruction on communication, team coordination, and environmental management; however, it is unclear whether and how these strategies are currently applied in EM residency training.^8^ In this study we aimed to assess the current state of resuscitation leadership training in EM residencies, examining the curriculum content and educational methods used in existing curricula.

## METHODS

### Study Design and Population

An anonymous, voluntary, needs assessment survey was distributed to ACGME-accredited EM residency program directors across the US from August–October 2021. We obtained contact information for program directors from the Society of Academic Emergency Medicine and the EMRA program databases. For programs with outdated or missing contact information, additional details were sourced from publicly available information on individual program websites. The survey was electronically administered using REDCap electronic data capture tools, hosted at William Beaumont University Hospital.^17^,^18^ The survey received institutional review board exemption from William Beaumont University Hospital.

**Population Health Research Capsule**

What do we already know about this issue?

*Resuscitation leadership improves patient outcomes; however, leadership training across graduate medical education is often limited*.

What was the research question?

*Do US emergency medicine residencies offer formal curricula for resuscitation leadership training?*

What was the major finding of the study?

*Of 80 (30.7%) responding EM residencies in the US, 19 (23.8%) reported offering a formal resuscitation leadership curriculum*.

How does this improve population health?

*Understanding current resuscitation leadership education can guide strategies to improve resuscitative care and outcomes across populations*.

### Survey Content

We designed the survey instrument to assess the presence and structure of formal resuscitation leadership curricula within EM residency programs in the US. Additionally, we aimed to explore the content and educational methods of these curricula and to determine whether programs relied on external resources for leadership training. The survey instrument was adapted from a previously published leadership needs assessment of US allopathic medical schools, developed by a group with extensive experience in medical education.^19^ For this study, we modified the instrument to shift the focus from medical school leadership education to EM residency resuscitation leadership education, with additional items to capture EM residency-specific demographics. The 10-question survey (Figure 1S in the supplement) solicited demographic information about the residency programs, the presence and structure of formal resuscitation leadership curricula, and the use of external leadership training resources. Resuscitation leadership was defined in the survey as “[t]he act of coordinating and motivating a team during a medical resuscitation of acutely, critically ill and decompensating individuals.” Notably, the survey focused exclusively on medical resuscitation education and excluded trauma resuscitation training.

### Statistical Analysis

We used descriptive analysis to summarize the findings from the needs assessment survey. After survey closure, we retrospectively conducted a post-survey wave analysis to detect potential non-response bias, a method previously used in health professions education research.^20^,^21^ We compared respondents to the first survey invitation with those responding after the final survey invitation using the Fisher exact test on the presence or absence of a formal resuscitation leadership curriculum. All statistical analyses were performed with SAS v9.4 (SAS Institute, Inc., Cary, NC).

## RESULTS

### Program Demographics

The needs assessment survey was distributed to 261 EM residency program directors in the US, with 80 responding (30.7%; AAPOR RR6).^21^ All US regions were represented among the respondents. Of the 80 programs, 63 (78.8%) were three-year and 17 (21.2%) were four-year residency programs. The majority of responding programs identified as academic (42 programs, 52.5%), while 30 programs (37.5%) were community-based, and eight programs (10.0%) identified as county-based.

### Resuscitation Leadership Curricula Characteristics

Of 80 responding programs, 19 residency programs (23.8%) reported offering a formal resuscitation leadership curriculum as part of their training. For these 19 programs all US regions were represented, with 14 (73.7%) three-year residencies and five (26.3%) four-year residencies. Ten programs were academic (52.6%), six community (31.6%), and three county (15.8%). Participation in the curriculum was mandatory for residents in all programs that offered it. Of the 19 residency programs with formal resuscitation leadership curricula, 10 programs (52.6%) implemented a longitudinal approach spanning postgraduate years (PGY) 1–3. Several programs opted to initiate training at more advanced stages of residency: two programs (10.5%) conducted training during PGY2–PGY3, and one program each (5.3%) conducted training during PGY 3–PGY 4, PGY 2 only, or PGY 3 only. One program (5.3%) reported conducting training exclusively during the PGY-1 year. In our wave analysis to measure potential non-response bias, there was no significant difference between early (N=35) and late (N=18) responders regarding the presence of a formal resuscitation leadership curriculum (*P* = 1.00).

Various educational methods were employed to deliver the curricula (Table 1), with simulation the most common approach (16 programs, 84.2%), followed by small-group discussions (12 programs, 63.2%), lectures (10 programs, 52.6%), and on-shift teaching (10 programs, 52.6%). Among the 19 programs with formal resuscitation leadership curricula, there were 17 unique combinations of educational methods employed (Table 1S). Notably, three programs (15.8%) used simulation as their sole educational method. The curriculum content also varied across programs (Table 2), with most focusing on clinical resuscitation leadership skills (18 programs, 94.7%); trauma resuscitation leadership skills (17 programs, 89.5%); communication and interpersonal skills (17 programs, 89.5%); and team building (13 programs, 68.4%). Programs reported 16 distinct combinations of curriculum content (Table 2S).

### External Leadership Programming

Of the 80 responding programs, 54 (67.5%) provided leadership education opportunities through external partnerships. These included hospital-sponsored leadership programs (30 programs, 37.5%), university-sponsored programs (17 programs, 21.3%), community-sponsored programs (10 programs, 12.5%), and research-focused leadership programs (14 programs, 17.5%). Nine programs (11.3%) reported offering other leadership education opportunities, including resources from contract groups, advocacy training, chief resident forums, and supplementary didactic sessions. Of the 19 programs with a formal resuscitation leadership curriculum, 13 (68.4%) offered additional leadership education opportunities through external partnerships.

## DISCUSSION

Prior studies have identified gaps in structured leadership training across graduate medical education. However, it remains unclear whether similar gaps exist within EM resuscitation leadership, an important consideration as the ACGME proposes additional resuscitation leadership requirements for EM residencies.^10^–^13^ To address this, we conducted a national needs assessment of EM residency programs to evaluate the presence of formal resuscitation leadership curricula.

Our assessment identified a gap in formal resuscitation leadership training among EM residency programs. We hypothesize that this deficiency is driven by several factors. A commonly cited challenge was the lack of time and resources within the crowded and complex medical education framework, where leadership and other “soft skills” are often deprioritized in favor of clinical and technical training.^22^ Learners also report barriers to leadership education, including difficulties in maintaining consistent leader-learner relationships, the lack of emphasis on leadership in feedback from superiors, and gender and racial dynamics that interfere with learning opportunities.^23^

In practice, much of the resuscitation leadership instruction that EM residents receive occurs through informal or “hidden” curricula. While many physicians do grow into effective resuscitation leaders within their clinical environments, literature from medical education broadly suggests that reliance on informal, or hidden, curricula can introduce variability, bias, and gaps in training.^1^,^24^,^25^ Additionally, EM residency leadership may assume that existing educational practices adequately develop residents’ leadership skills in resuscitations, reducing the perceived need for formal curricula. However, no studies have rigorously evaluated whether current approaches consistently produce graduates with the necessary skills. Further research is needed to determine whether existing training sufficiently prepares EM residents to competently lead emergent resuscitations.

An additional factor that contributes to the absence of high-quality resuscitation leadership curricula is the variability in resuscitation leadership training across medical education. Our assessment identified diversity in resuscitation leadership content and educational methods across programs. Simulation-based training was the most frequently used method, which aligns with its widespread adoption in EM residency education.^26^ We hypothesize that its prominence in resuscitation leadership education reflects the unique strengths of simulation in replicating high-stakes resuscitations within a controlled learning environment. Additionally, simulation provides structured opportunities to practice and receive feedback on resuscitation leadership skills that are more difficult to convey through didactics or clinical shifts alone.^27^,^28^ Further analysis of best practices for resuscitation leadership educational methodology is needed to determine the best form of instruction for EM residents.

Commonly identified content themes included leadership performance, communication, and team-building. Notably, many programs reported incorporating both medical and trauma leadership skills into their curricula. We suspect this reflects an effort to build transferable leadership competencies across different resuscitation contexts. However, the degree to which trauma-focused leadership training complements or enhances medical resuscitation leadership skills remains unclear. Importantly, no program reported trauma leadership as the sole focus of its curriculum. Our findings also highlight substantial variability in curriculum content across programs, mirroring broader inconsistencies in leadership training across graduate and undergraduate medical education, as reported by Matsas et al and Rosenman et al. 29,30 Both reviews highlighted the lack of standardized leadership frameworks as a key contributor to the slowed progress of leadership curriculum development and likely impacted the development of content specific to resuscitation leadership. Further research is needed to identify best practices and to evaluate how different curriculum content shapes EM residents’ resuscitation leadership skills.

Within EM, the Milestones Project offers the closest thing to a resuscitation leadership competency framework, but its lack of specificity hinders its application for developing resuscitation leadership curricula as indicated by Rosenman et al.^8^ Furthermore, the milestones do not cover key administrative, management, and conflict-resolution skills expected of practicing emergency physicians during resuscitations. Our study reflects previously reported gaps in opportunities for EM residents to develop these broader resuscitation leadership competencies.^14^ The development of consensus, competency-based frameworks for resuscitation leadership within EM warrants further investigation.

There is a wealth of validated leadership models in fields such as business, the military, and aviation that could serve as valuable resources for EM trainees.^15^ Many responding EM residency programs provide voluntary leadership training through external partnerships, including hospital, university, and community-based programs, which may draw on these external leadership models. Programs like Crew Resource Management, US Army officer leadership programs, and executive leadership courses have shown success in improving leadership skills.^10^,^15^ Similarly, government-sponsored healthcare leadership programs such as TeamSTEPPS and STARTT have demonstrated promise in enhancing leadership capabilities.^31^,^32^ Non-EM specialty resuscitation leadership curricula have also been explored and show significant improvements in patient outcomes in pediatric, traumatic and intensive care unit resuscitations.^33^–^37^ Additionally, research programs focused on resuscitation leadership may contribute relevant leadership skills.

Emergency medicine residents are also expected to complete standardized, guideline-based training including Advanced Trauma Life Support (ATLS), Advanced Cardiovascular Life Support (ACLS), and Pediatric Advanced Life Support, which include elements relevant to both trauma and medical resuscitations. However, it remains unclear how effectively these external models can be adapted to the unique demands of medical resuscitation leadership in EM.^33^ For example, while ATLS may incorporate some leadership principles, the degree to which it or similar courses develop the real-time leadership skills required for leading diverse emergency teams is uncertain. Notably, prior research has shown that ACLS training alone is insufficient to prepare physicians for resuscitation leadership roles.^5^ Further, the variability in content and delivery across these programs raises questions about which approaches are best suited for EM residency training. Future research should focus on exploring how these external leadership frameworks could be tailored to EM resuscitation leadership education. Moreover, addressing the barriers that prevent the implementation of resuscitation leadership training in EM residencies will be key to developing and integrating effective programs in the future.

## LIMITATIONS

Our study has limitations that should be acknowledged. The overall response rate was relatively low, which increases the risk of non-response bias as non-respondents may have had different needs in resuscitation leadership then respondents. Additionally, our study was underpowered to detect resuscitation leadership curriculum differences in three- vs four-year EM residency programs. However, our wave analysis demonstrated no significant difference between early and late responders, suggesting that non-respondent data would be unlikely to substantially alter the results.^21^ Moreover, the proportion of three- vs four-year programs among respondents closely paralleled national distributions at the time of the survey.^38^ Geographic representation was also broadly similar, with modest over-representation of Great Lakes programs and under-representation of Great Plains programs.

Our survey instrument did not undergo formal validity testing or piloting, which may have led to variable interpretation of survey items. In particular, “on-shift teaching” was listed as one of several educational methods by programs that reported having a formal curriculum; however, we recognize that the structure and consistency of this approach may vary across institutions. Lastly, survey responses were taken from program leadership perspective and lack resident insights on the impact of resuscitation leadership training. However, EM resident lack of leadership training has been well documented previously, and we assumed that such a gap likely extends to resuscitation leadership.

## CONCLUSION

Most of the US EM residency programs that participated in this study reported lack of a formalized resuscitation leadership curriculum, with existing programs showing considerable variability in educational methods and curriculum content. This inconsistency mirrors broader challenges in leadership education across medical training. Additional research is essential to determine whether traditional leadership training improves resuscitation performance and patient outcomes, and to inform the potential development of targeted educational strategies. While there is potential to adapt established leadership programs from other fields to emergency medicine, further research is necessary to validate their effectiveness in enhancing resuscitation leadership skills.

## Supplementary Information



## Figures and Tables

**Table 1:** Resuscitation leadership curriculum content delivery methods for 19 residency programs.

Curriculum delivery	Frequency (n)	Percentage (%)
Lectures	10	52.63
Small-group discussions	12	63.16
Seminars/Workshops	1	5.26
Simulation	16	84.21
Case studies	5	26.32
Self-directed learning	2	10.53
On-shift teaching	10	52.63
Mentorship	7	36.84
Journal Club	1	5.26

**Table 2:** The focus of resuscitation leadership curriculum content in 19 residency programs.

Curriculum focus	Frequency (n)	Percentage (%)
Clinical resuscitation leadership skills	18	94.74
Trauma resuscitation leadership skills	17	89.47
Administrative leadership skills	1	5.26
Communication and interpersonal skills	17	89.47
Cultural sensitivity	3	15.79
Teaching/education	5	26.32
Health policy and managed care	0	0
Leadership theory	6	31.58
Team-building	13	68.42
Management skills	7	36.84
Conflict resolution	8	42.11
