# Supplementary material for: Resuscitation Leadership Education: A Needs Assessment of Emergency Medicine Residencies
Source: West J Emerg Med. 2025 Dec 26;27(1):33–8. doi: 10.5811/westjem.47285 (PMC12815503; doi:10.5811/westjem.47285)
Supplement: Supplementary file 1 [file wjem-27-33-s001.pdf]

**Figure 1S.** Program Director Survey

Thank you for taking the time to fill out this brief program director survey on medical resuscitation leadership initiative (MERLIN). For the sake of this survey, resuscitation leadership will be defined as: The act of coordinating and motivating a team during resuscitation of acutely critically ill and decompensating individuals. Please note that this **does not** include critically ill trauma patients. Your participation in this survey is voluntary. By submitting this survey, you voluntarily agree to have your responses recorded. Your identity is not shared as part of submitting this survey. This survey was adapted from Neely, Clyn, &Resinick-Ault.[1]

1. Residency Program Geographic Location
    a. Pacific
    b. Southwest
    c. Great Plains
    d. Great Lakes
    e. South Central
    f. Southeast
    g. Mid-Atlantic
    h. Northeast
2. Length of program
    a. 3 years
    b. 4 years
3. Program Type
    a. Academic
    b. Community
    c. County
4. Does your residency program offer a formal resuscitation leadership curriculum?
    *a. If no, continue to question 10*
5. Is participation in the resuscitation leadership curriculum required or elective?
    a. Required
    b. Elective
    c. Other
6. What percentage of residents participate in the leadership training curriculum?
    a. 0-25%
    b. 26-50%
    c. 51-75%
    d. 76-100%
7. How is the resuscitation leadership education delivered? Select all that apply
    a. Lectures
    b. Small Group Discussions
    c. Seminars/Workshops
    d. Simulation
    e. Case studies
    f. Self-directed learning
    g. On-shift teaching
    h. Mentorship

   i. Journal Club
   j. Other
8. At what point of training do residents participate in the resuscitation leadership curriculum? Select all that apply
   a. PGY 1
   b. PGY 2
   c. PGY 3
   d. PGY 4
9. Please briefly describe your program's resuscitation leadership curriculum focuses. Select all that apply
   a. Clinical Resuscitation Leadership Skills
   b. Trauma Resuscitation Leadership Skills
   c. Administrative Leadership skills
   d. Communication & Interpersonal skills
   e. Cultural sensitivity
   f. Teaching/education
   g. Health policy and managed care
   h. Leadership theory
   i. Team building
   j. Management skills
   k. Conflict resolution
   l. Other
10. Are there any additional opportunities for residents to participate in leadership programs through your residency's partnerships? Select all that apply
   a. Hospital Sponsored program
   b. University Sponsored Program
   c. Community Sponsored Program
   d. Research Opportunities
   e. Other

**Table 1S.** Distribution of educational methods used in EM residency resuscitation leadership curricula (n=19)

| Lectures | Small Group Discussions | Seminars/ Workshops | Simulation | Case Studies | Self-directed Learning | On-shift teaching | Mentorship | Journal Club | Other | Frequency | Percent |
|---|---|---|---|---|---|---|---|---|---|---|---|
| . | . | . | X | . | . | . | . | . | . | 3 | 15.79 |
| . | . | . | X | . | . | X | X | . | . | 1 | 5.26 |
| . | X | . | X | . | . | . | . | . | . | 1 | 5.26 |
| . | X | . | X | . | . | X | . | . | . | 1 | 5.26 |
| . | X | . | X | X | . | . | . | . | . | 1 | 5.26 |
| . | X | . | X | X | . | X | X | . | . | 1 | 5.26 |
| . | X | X | X | . | . | . | X | . | . | 1 | 5.26 |
| X | . | . | . | . | . | X | . | . | . | 1 | 5.26 |
| X | . | . | X | . | . | X | X | . | . | 1 | 5.26 |
| X | . | . | X | X | . | X | . | . | . | 1 | 5.26 |
| X | X | . | . | . | . | X | X | . | . | 1 | 5.26 |
| X | X | . | . | X | . | X | . | . | . | 1 | 5.26 |
| X | X | . | X | . | . | . | . | . | . | 1 | 5.26 |
| X | X | . | X | . | . | X | . | . | . | 1 | 5.26 |
| X | X | . | X | . | . | X | X | X | . | 1 | 5.26 |
| X | X | . | X | . | X | . | X | . | . | 1 | 5.26 |
| X | X | . | X | X | X | . | . | . | . | 1 | 5.26 |

**Table 2S.** Distribution of curriculum content for EM residency resuscitation leadership curricula (n=19)

| Clinical Resuscitation | Trauma Resuscitation | Administrative | Communication & Interpersonal | Cultural sensitivity | Teaching/ education | Health Policy & managed care | Leadership Theory | Team Building | Management skills | Conflict resolution | Other | Frequency | Percent |
|---|---|---|---|---|---|---|---|---|---|---|---|---|---|
| . | X | . | X | . | . | . | . | X | . | . | . | 1 | 5.26 |
| X | . | . | X | . | . | . | . | X | . | . | . | 1 | 5.26 |
| X | . | . | X | . | . | . | . | X | . | X | . | 1 | 5.26 |
| X | X | . | . | . | . | . | . | . | . | . | . | 2 | 10.53 |
| X | X | . | X | . | . | . | . | . | X | . | . | 1 | 5.26 |
| X | X | . | X | . | . | . | . | X | . | . | . | 1 | 5.26 |
| X | X | . | X | . | . | . | . | X | . | X | . | 1 | 5.26 |
| X | X | . | X | . | . | . | . | X | X | . | . | 3 | 15.79 |
| X | X | . | X | . | . | . | X | . | . | . | . | 1 | 5.26 |
| X | X | . | X | . | . | . | X | X | . | X | . | 1 | 5.26 |
| X | X | . | X | . | X | . | . | . | . | . | . | 1 | 5.26 |
| X | X | . | X | . | X | . | X | X | X | X | . | 1 | 5.26 |
| X | X | . | X | X | . | . | X | . | . | X | . | 1 | 5.26 |
| X | X | . | X | X | X | . | . | X | X | X | . | 1 | 5.26 |
| X | X | . | X | X | X | . | X | X | . | X | . | 1 | 5.26 |
| X | X | X | X | . | X | . | X | X | X | X | . | 1 | 5.26 |
